# Carers’ needs assessment for patients with dementia in Ghana

**DOI:** 10.4102/phcfm.v14i1.3595

**Published:** 2022-08-22

**Authors:** Nana K. Ayisi-Boateng, Douglas A. Opoku, Phyllis Tawiah, Ruth Owusu-Antwi, Emmanuel Konadu, Georgina T. Apenteng, Akye Essuman, Charles Mock, Bernard Barnie, Peter Donkor, Fred S. Sarfo

**Affiliations:** 1 Department of Medicine, School of Medicine and Dentistry, Kwame Nkrumah University of Science and Technology, Kumasi, Ghana; 2 School of Public Health, Kwame Nkrumah University of Science and Technology, Kumasi, Ghana; 3 Department of Behavioural Sciences, School of Medicine and Dentistry, Kwame Nkrumah University of Science and Technology, Kumasi, Ghana; 4 University Hospital, Kwame Nkrumah University of Science and Technology, Kumasi, Ghana; 5 Department of Family Medicine, University Hospital, Kwame Nkrumah University of Science and Technology, Kumasi, Ghana; 6 Department of Family Medicine, University of Health and Allied Sciences, Ho, Ghana; 7 Department of Epidemiology, School of Public Health, University of Washington, Washington, United States; 8 Department of Surgery, School of Medicine and Dentistry, Kwame Nkrumah University of Science and Technology, Kumasi, Ghana

**Keywords:** carers, dementia, Ghana, intervention, needs, problem area

## Abstract

**Background:**

Carers of people with dementia (PWD) face a myriad of challenges. As dementia prevalence surges in the sub-Saharan population, the provision of data on the met and unmet needs of caregivers has become paramount.

**Aim:**

This study aimed to identify the needs of carers of older adults living with dementia in Ghana.

**Setting:**

This study was conducted in Kumasi, Ghana, among participants ≥ 18 years old, serving as carers for PWD.

**Methods:**

This was a multicentre cross-sectional study involving carers of patients (≥ 50 years) with dementia. The authors administered the Carer’s Needs Assessment for Dementia (CNA-D) questionnaire, containing 18 problem areas with interventions for each problem area. Pearson’s correlation analysis was performed to establish a relationship between demographic characteristics, problem areas and intervention score.

**Results:**

Fifty participants were recruited with a mean age of 48.8 (± 16.9) years, 72.0% were female participants and 98.0% were family members of PWD. The problem area most frequently identified as no/mild problem was ‘legal issues’ (92.0%, *n* = 46), and ‘lack of information about dementia’ was assessed as severe (48.0%, *n* = 24). The commonest unmet need was ‘printed information material’ (84.0%, *n* = 42), and the commonest met need was ‘diagnosis and treatment of carer by a general practitioner’ (42.0%, *n* = 21). There was a statistically negative correlation between age of carer and number of unmet needs (*r* = −0.308, *p* = 0.011) and a positive correlation between problem area score and number of unmet needs (*r* = 0.308, *p* = 0.030).

**Conclusion:**

Effective public education and provision of information on dementia to carers are essential interventions needed to equip them in performing their roles.

**Contribution:**

Carers in this study revealed that they lacked information on dementia but their commonest met need was accessibility to their general practitioner. This highlights the importance of promoting knowledge and awareness of dementia among primary care practitioners.

## Introduction

Dementia is characterised by the significant neurocognitive disorder that may affect the language (aphasia), ability to perform motor activities (apraxia), ability to recognise objects (agnosia), mood, personality, behaviour and performance of activities of daily living.^1^ Globally, more than 50 million people are living with dementia with an annual increase of 10 million new cases.^1^ Approximately 60% of these are living in low- and middle-income countries, in spite of the relatively low average life expectancy among this population.^1,2^

The pooled crude prevalence of dementia is 4.9% (95.0% confidence interval [CI]: 3.0–6.9) in Nigeria alone and 2.4% in Africa as a whole, with Alzheimer’s disease rated as the most prevalent cause (57.1%) followed by vascular dementia (26.9%).^2,3,4^ In Ghana, approximately 50.0% of stroke survivors have been found to have cognitive dysfunction and 13.6% have vascular dementia.^5^

Caring for people with dementia (PWD) can be demanding and emotionally, mentally, financially and physically taxing. Family members often experience anxiety, fears, depressive moods and stigma. There are a number of tools devised to assess the needs of carers of PWDs, one of which is the Carers’ Needs Assessment for Dementia (CNA-D).^6^ The CNA-D questionnaire has been employed and validated in other populations,^7,8^ but rarely in an African population. Generally, there is a paucity of research-generated data on dementia in Africa. Challenges associated with dementia-related studies include stigma resulting in the reluctance of family members to present their affected relatives for care, the belief that an elderly person has completed their useful life, differential survival rates, variations in case detection tools and errors in their interpretation, and the belief that dementia is a normal part of ageing.^9^ Little is known about the challenges and needs of carers or families living with sufferers of dementia.

The authors, therefore, sought to assess the met and unmet needs of carers of PWD. The authors explored their feelings such as guilt, fears, social withdrawal or stigma and assessed their knowledge on information about dementia, diagnosis, financial aids and available services.

## Methods

### Study design and population

This was a cross-sectional study. Participants were carers of patients who were at least 50 years old and previously diagnosed with dementia. One carer or family member per patient was recruited, and these were individuals, 18 years and above, who spent at least one physical contact per day with the patient. The study excluded carers of patients with any comorbid psychiatric or neurological illness such as schizophrenia, bipolar disorder or major depression. Patients with other conditions such as chronic kidney disease, decompensated liver disease and vitamin B12 deficiency, which may cause cognitive impairment, were also excluded. All recruited participants had to demonstrate an ability to understand the study and a willingness to provide informed consent.

### Study area

This was a multicentre study conducted at four clinics, namely, the Neurology Clinic and Psychiatry Unit at the Komfo Anokye Teaching Hospital (KATH), the Family Medicine Clinic and Geriatric Clinic at the University Hospital, Kwame Nkrumah University of Science and Technology (KNUST), and the Centre for Ageing and Elderly Care at Metro Health Hospital. All three hospitals are located in Kumasi, an urban area in the Ashanti Region of Ghana. Komfo Anokye Teaching Hospital is a 1200-bed tertiary-level hospital, and its Neurology Clinic was set up in the year 2011.^10^ Approximately 50.0% of stroke survivors at the clinic have cognitive dysfunction and 13.6% have vascular dementia. The Psychiatry Unit at KATH was also set up in 1981 and runs a weekly neuropsychiatric clinic for the treatment of patients with dementia of Alzheimer’s type.

The University Hospital, KNUST, is a 125-bed district-level hospital that provides general and specialist services for staff of the University and their dependants, students and other private patients, within the Kumasi Metropolis. The Family Medicine Clinic and Geriatric Clinic at KNUST are run once a week. The Centre for Ageing and Elderly Care at Metro Health Hospital, a privately owned primary care facility that was commissioned in early 2020, has been managing patients with dementia under the care of a geriatrician, but there is no readily available data on the number of cases seen by type. Likewise, prior to the conduct of this study, data were lacking on patients with dementia who are seen at the Department of Psychiatry, KATH, Family Medicine Clinic and Geriatric Clinic at the University Hospital, KNUST.

### Sample size calculation

The sample size was calculated using the formula (Eqn 1):


$$\text{Sample size}=\frac{{\text{Z}}^{2}(\text{P})(1-\text{P})}{E^{2}}$$ [Eqn 1]

Based on an anticipated prevalence of 15% for specific unmet needs,^6^ 10% allowable margin of error and 95% CI, the estimated sample size was 49 participants. Hence, a total of 50 participants were recruited.

### Sampling process

Participants were consecutively sampled from patients (≥ 50 years old) with history of dementia diagnosed at the KATH, Metro Health Hospital and the University Hospital, KNUST. Two research assistants of this study conducted a search of the manual and electronic patient records at the three facilities to identify patients who have a previous diagnosis of dementia. Those with available phone numbers or traceable addresses were contacted either on the phone or a nurse at the clinics assisted in going to the patients’ homes. The aim and objectives of the study were explained to them. Only caregivers who fitted the inclusion criteria and provided an informed consent were recruited into the study. Participants were recruited over a four-month period from July to November 2021.

### Data collection and analysis

The medical information of all relevant patients was retrieved from the medical records at the respective study sites. Demographic data that were missing from the medical records were obtained from the carer or the patient. A standardised questionnaire that included the CNA-D tool was administered to all participants. Prior to the administration of the questionnaire, it was translated from English to Twi (the local language) and back-translated by language experts from the Ghana Institute of Languages. It was pretested among 10 carers whose data were not included in the final analysis. Data obtained from the recruited participants included the socio-economic, demographic and clinical characteristics of caregivers, as well as the patients with dementia. The CNA-D is a semi-structured interview guide that assesses caregiver needs across 18 problem areas and interventions for each problem area. The CNA-D is designed to determine the need for intervention using a 5-point Likert-type scale from 0 = no need (intervention not needed and not received), 1 = over provision (intervention was not needed but received), 2 = unmet need (intervention was needed and not received), 3 = partially met need (intervention was needed and insufficiently received) to 4 = met need (intervention needed and sufficiently received).^6^

For each category of responses under problem areas and intervention, the authors highlighted those that had the highest proportion of carers. Data validation procedure was undertaken to improve the accuracy and quality of the data. Data were exported to STATA version 16 for analysis and were presented as frequencies, percentages and means. The severity of the problem for a participant was rated as no/mild problem = 0, moderate problem = 1, and severe problem = 2. The problem scores were computed for each participant. The total number of unmet and met needs was rated by counting the number of unmet needs and met needs, respectively, by a participant. The intervention score was rated as no need/overprovision/partially met need/met need = 0 and unmet need = 1. Similarly, the met needs score was rated as no need/overprovision/partially met need/unmet need = 0 and unmet need = 1. Pearson’s correlation analysis was performed to establish a relationship between demographic characteristics and problem area score, as well as intervention score (unmet needs and met needs). All values with *p* ≤ 0.05 were reported as statistically significant at a 95.0% CI.

### Ethical considerations

Ethical approval to conduct the study was obtained from the Institutional Review Board (IRB) of the Komfo Anokye Teaching Hospital (KATH), Kumasi, Ghana (No. KATH IRB/ AP/013/21).

## Results

### Demographic characteristics of the carers and patients with dementia

The mean (± s.d.) age of the carers was 48.8 (± 16.9) years with a minimum age of 20 years and a maximum age of 87 years. Approximately 72.0% (*n* = 36) of the carers were females, 62.0% (*n* = 31) of them were married and 38.0% (*n* = 19) had basic education. About 20.0% (*n* = 10.0) of the carers had hypertension. Of the 50 recruited caregivers, 49 (98.0%) were family members of the PWD. The minimum duration a study participant had been engaged in caring for a PWD was 6 months and the maximum was 28 years, with a median duration of 2 years. The mean number of hours per day spent with a PWD was 12.3 (± 6.3) h with a minimum of 2 h and a maximum of 24 h (Table 1).

**TABLE 1:** Demographic characteristics of the carers.

Variables	Frequency (*N* = 50)	Percentage (%)
**Age of carer (years)**		
20–29	9	18.0
30–39	7	14.0
40–49	10	20.0
50–59	11	22.0
60+	13	26.0
**Sex**		
Male	14	28.0
Female	36	72.0
**Marital status**		
Single	16	32.0
Married	31	62.0
Divorced or separated	3	6.0
**Level of education**		
Basic	19	38.0
Secondary	18	36.0
Tertiary	10	20.0
None	3	6.0
**Religion**		
Christian	47	94.0
Muslim	3	6.0
**Employment status**		
Formal	9	18.0
Informal	17	34.0
Self-employed	5	10.0
Unemployed	19	38.0
**Comorbidity of caregiver**		
Hypertension	10	20.0
Stroke	1	2.0
None	39	78.0
**Number of children**		
0–2	23	46.0
3–5	21	42.0
6+	6	12.0
**Payment for care**		
Yes	4	8.0
No	46	92.0
**Number of hours spent with patient in a day**		
< 12	19	38.0
≥ 12	31	62.0
**Years of engagement**		
< 1	14	28.0
1–5	28	56.0
> 5	8	16.0
**Number of people taking care of patient aside carer**		
0	13	26.0
1–2	33	66.0
≥ 3	4	8.0
**Relationship with patient**		
Family member†	49	98.0
Worker (hired)	1	0.2

Note: Age of carer (years): Mean = 48.8, s.d. = ± 16.9, range = 20–87. Number of hours spent with patient in a day: Mean = 12.3, s.d. = ± 6.3, range = 2–24. Years of engagement: Median duration = 2, range = 0.6, 28.0.

† , Includes son, daughter, mother, uncle, brother, sister and grandchild.

The mean age of the patients with dementia was 76.2 years (± 10.1) with a minimum age of 50 years and a maximum age of 95 years. About 56.0% (*n* = 28) of the patients with dementia were females. Approximately 86.0% (*n* = 43) of the patients had a diagnosis of vascular dementia. Hypertension (44.6%, *n* = 33) was the commonest comorbidity among patients with dementia (Table 2).

**TABLE 2:** Demographic and clinical characteristics of patients with dementia.

Variables	Frequency (*N* = 50)	Percentage (%)
**Sex of patient**		
Male	22	44.0
Female	28	56.0
**Dementia type**		
Alzheimer	7	14.0
Vascular dementia	43	86.0
**Duration of illness (years)**		
< 1	12	24.0
1–5	29	58.0
> 5	9	18.0
**Comorbidity of patient†**		
Hypertension	33	44.6
Diabetes	18	24.3
Stroke	10	13.5
Asthma or COPD	4	5.4
None	9	12.2
**Distribution of participants at study sites**		
Komfo Anokye Teaching Hospital	30	60.0
University Hospital	4	8.0
Metro Health Hospital	16	32.0

Note: Age of patient: Mean = 76.2, s.d. = ± 10.1, range = 50–95. Durarion of illness (years): Mean = 3.7, s.d. = ± 4.4, range = 0.6–28.

COPD, comorbidity among patients with dementia.

† , Multiple responses.

### Descriptive statistics of problem area score, met needs and unmet needs

The median problem score of a participant was 12.0 with a minimum problem score of 0 and a maximum problem score of 32. The median total number of met needs was 2.0 with a minimum number of met needs of 0 and a maximum number of met needs of 8. The median total number of unmet needs was 12.0 with a minimum number of unmet needs of 0 and a maximum number of unmet needs of 18 (Table 3).

**TABLE 3:** Descriptive statistics of problem area score, met needs and unmet needs.

Variables	Median	IQR	Minimum	Maximum
Problem areas score	12.0	11.0	0	32
Number of met needs	2.0	2.0	0	8
Number of unmet needs	12.0	8.0	0	18

IQR, interquartile range.

### Problem areas and severity assessment by carers

Generally, most carers assessed the problem areas as no problem or mild problem. In descending order, problem areas with the highest frequency assessed as no/mild by carers were ‘legal issues’ (92.0%, *n* = 46), ‘fear of stigmatisation’ (82.0%, *n* = 41), ‘feelings of guilt, being blamed’ (82.0%, *n* = 41), ‘physical or psychiatric illness of carer’ (78.0%, *n* = 39), ‘conflicts with family’ (68.0%, *n* = 34), ‘missing nursing skills’ (64.0%, *n* = 32), ‘social isolation’ (62.0%, *n* = 31) and ‘lack of information about services’ (52.0%, *n* = 26).

Problem areas with the highest frequency assessed as severe by the carers were ‘lack of information about dementia’ (48%, *n* = 24) followed by ‘problems caused by crisis’ (46.0%, *n* = 23) and ‘lack of information about treatment’ (44.0%, *n* = 22) (Table 4).

**TABLE 4:** Problem area and severity assessment.

Problem area	Rating of problem area (*N* = 50)					
	None to mild		Moderate		Severe	
	*n*	%	*n*	%	*n*	%
Lack of information about dementia	19	38.0	7	14.0	24	48.0
Lack of information about treatment	14	28.0	14	28.0	22	44.0
Lack of information about services	26	52.0	16	32.0	8	16.0
Financial burden	18	36.0	14	28.0	18	36.0
Legal issues	46	92.0	2	4.0	2	4.0
Disappointment caused by the illness, concerns about the patient’s future	23	46.0	6	12.0	21	42.0
Communication problems and conflicts with the patient	25	50.0	9	18.0	16	32.0
Burden caused by behavioural problems of patients	24	48.0	7	14.0	19	38.0
Problems caused by crisis	23	46.0	4	8.0	23	46.0
Not enough time for oneself (including caring for the patient when the relative becomes sick)	19	38.0	13	26.0	18	36.0
Social isolation	31	62.0	8	16.0	11	22.0
Conflicts with family	34	68.0	4	8.0	12	24.0
Burden caused by dangerous situations	23	46.0	11	22.0	16	32.0
Fear of stigmatisation	41	82.0	4	8.0	5	10.0
Feelings of guilt, being blamed	41	82.0	5	10.0	4	8.0
Missing nursing skills	32	64.0	11	22.0	7	14.0
Difficulties concerning household tasks	38	76.0	5	10.0	7	14.0
Burned out or overstrained by care	24	48.0	14	28.0	12	24.0
Physical or psychiatric illness of the carer	39	78.0	8	16.0	3	6.0

### Frequency of interventions selected by carers as appropriate for solving one or more problems

The commonest unmet need (intervention) among the respondents was ‘printed information material’ (84.0%, *n* = 42) followed by ‘hotline where the carer can get advice in crises’ (82.0%, *n* = 41). Other unmet needs were ‘training of practical skills for the carer (e.g. basic nursing skills)’ (78%, *n* = 39), ‘support from a social worker’ (74.0%, *n* = 37), ‘mobile nursing care for outpatients’ (72%, *n* = 36), ‘self-help group for family members’ (68.0%, *n* = 34), ‘temporary supervision of the patient at home’ (66.0%, *n* = 33), ‘individual psychoeducation’ (64.0%, *n* = 32), ‘group psychoeducation’ (60.0%, *n* = 30), ‘relatives group guided by a professional’ (56.0%, *n* = 28), ‘care for the patient in a day centre’ (56.0%, *n* = 28) and ‘financial compensation’ (52.0%, *n* = 26) (Table 5).

**TABLE 5:** Interventions and frequency of carers’ assessment of met and unmet needs.

Intervention	No need		Overprovision		Unmet need		Partially met need		Met need	
	*n*	%	*n*	%	*n*	%	*n*	%	*n*	%
Counselling and emotional support	9	18.0	1	2.0	23	46.0	5	10.0	12	24.0
Relatives group guided by a professional	8	16.0	0	0.0	28	56.0	5	10.0	9	18.0
Self-help group for family members	8	16.0	0	0.0	34	68.0	5	10.0	3	6.0
Individual psychoeducation	11	22.0	1	2.0	32	64.0	3	0.6	3	0.6
Group psychoeducation	15	30.0	2	4.0	30	60.0	2	4.0	1	2.0
Printed information material	6	12.0	1	2.0	42	84.0	1	2.0	0	0.0
Support from a social worker	10	20.0	0	0.0	37	74.0	2	4.0	1	2.0
Financial compensation	6	12.0	3	6.0	26	52.0	11	22.0	4	8.0
Temporary supervision of the patient at home	8	16.0	0	0.0	33	66.0	3	6.0	6	12.0
Care for the patient in a day centre	20	40.0	0	0.0	28	56.0	0	0.0	2	4.0
Mobile personal care for outpatients	9	18.0	0	0.0	31	62.0	5	10.0	5	10.0
Training of practical skills for the carer (e.g. basic nursing skills)	3	6.0	0	0.0	39	78.0	3	6.0	5	10.0
Diagnosis or treatment of the carer by a general practitioner	3	6.0	0	0.0	23	46.0	3	6.0	21	42.0
Mobile nursing care for outpatients	8	16.0	0	0.0	36	72.0	2	4.0	4	8.0
Holidays together with the patient in a specialised setting	21	42.0	0	0.0	24	48.0	1	2.0	4	8.0
Respite care (short-term relief for carer)	5	10.0	1	2.0	18	36.0	11	22.0	15	30.0
General assistance for household chores	14	28.0	2	4.0	11	22.0	5	10.0	18	36.0
Hotline where the carer can get advice in crises	1	2.0	0	0.0	41	82.0	3	6.0	5	10.0
Social contact centre	20	40.0	0	0.0	26	52.0	3	6.0	1	2.0

Among the met needs, the one with the highest proportion of carers was ‘diagnosis or treatment of carer by a general practitioner’ (42%, *n* = 21) followed by ‘general assistance for household chores’ (36.0%, *n* = 18). None of the interventions recorded a higher percentage of participants assessing it as ‘no need’, ‘overprovision’ or ‘partially met’ (Table 5).

### Correlation of demographic characteristics, problem area score, and met and unmet needs

There was a statistically negative correlation between the age of a carer and the number of unmet needs (*r* = –0.356, *p* = 0.011). The number of unmet needs of a carer decreases with increasing age. There was also a statistically positive correlation between a problem area score and the number of unmet needs (*r* = 0.308, *p* = 0.030). That is, the problem area score increases with increasing unmet needs (Table 6).

**TABLE 6:** Correlation of demographic characteristics, problem area score, and met and unmet needs.

Variables	Problem		Unmet need		Met needs	
	*r*	*p*	*r*	*p*	*R*	*p*
Age of carer (years)	−0.047	0.744	−0.356	0.011†	0.162	0.261
Sex	−0.164	0.254	−0.237	0.097	0.180	0.211
Relationship status	0.009	0.949	−0.103	0.476	0.170	0.238
Level of education	−0.188	0.191	−0.116	0.422	−0.047	0.748
Employment status	0.027	0.853	−0.230	0.107	0.166	0.250
Number of children	0.119	0.410	−0.231	0.106	0.171	0.234
Payment for care	−0.049	0.736	0.030	0.836	−0.079	0.584
Number of hours spent with patient in a day	0.045	0.754	−0.040	0.785	0.045	0.757
Years of engagement	−0.096	0.506	−0.215	0.134	0.167	0.247
Problem area score	-	-	0.308†	0.030	−0.276	0.052

*r*, Correlation coefficient.

† , Correlation is significant at the 0.05 level (two-tailed).

## Reliability

The Cronbach’s coefficient (internal consistency) for the ‘summary score’ of problem areas reported by the carers was 0.86. The ‘summary score’ for the interventions as reported by the carers was 0.74. These values indicate that among these respondents, the CNA-D questionnaire was a highly reliable tool for assessing the carers’ needs and suggested interventions.

## Discussion

### Demographic characteristics of carers

Dementia, as a chronic debilitating illness, places a huge burden on the patient and carers, some of whom may be family members. The problems they face, as well as the needed interventions, may be influenced by sociocultural, socio-demographic and geographical factors. In the study, the mean age of the carers was 48.8 (± 16.9) years, comparable with a study in Europe in which the carers had a mean age of 50 years, whereas 66.7 years (± 16.1) was reported elsewhere.^11^ Approximately 72.0% of the participants were females, consistent with global trends that show a high female caregiver preponderance.^12,13,14^ It is also reported that three-quarters of PWD receive care in the community, with the majority of the carers being females.^12^ While this signifies the supportive role females play at homes in Ghana and other parts of the world, men caregivers increased by 21.0% in the United States between 1996 and 2008, and in the United Kingdom, men older than 75 years are more likely than women to be carers of their spouse.^15,16^

Family carers are considered ‘invisible second patients’ and comprised 98.0% of the participants.^12^ However, the authors did not look at the proportion of relatives that were spouses, children, nieces, nephews or in-laws. Earlier studies showed that over half of carers were relatives of the patients (either son or daughter) or spouses who had chosen to take care of the patients in their own homes.^17,18^ In China, almost half of dementia care was provided by the patients’ children or children-in-law (47.7%), whereas sons were four times more likely than daughters (81.0% vs. 19.0%, *p* < 0.001) to provide informal care.^19^ These findings emphasise the role of family carers in the lives of PWD in both resource-limited settings and in places where nursing homes are more readily available.

### Demographic and clinical characteristics of patients with dementia

In this study, the mean age of PWD was 76.2 years (± 10.1), comparable with 73 years (65–110) among 12 865 patients recruited in a global study on dementia.^20^ Age is an important risk and prognostic factor for dementia^21^ in both males and females. About 56.0% of the PWD in the study were females in consonance with previous studies. Data from North America and Europe show a greater prevalence of dementia among women than men.^22,23^ This has often been attributed to the relatively longer lifespan of women.^24^ Women have also been found to perform significantly worse than men across most cognitive domains, possibly because of the lower level of educational attainment, poorer early-life nutrition and other gender socio-economic disparities.^24^ These adversely impact the brain health and cognitive ability of women as they age. In spite of these, gender differences have not been found to be a statistically significant risk factor for developing dementia.^25,26^ Further studies are required to ascertain whether the above assertions are reproducible in the African population.

Among the 50 patients, 43 had a diagnosis of vascular dementia with the other 7 having Alzheimer’s disease. However, the study that was hospital-based contrasts with findings in an earlier community-based study that reported Alzheimer’s disease as the most prevalent cause of dementia (57.1%) followed by vascular dementia (26.9%).^27^ Vascular dementia is also generally considered the second most common subtype of dementia, after Alzheimer’s disease, accounting for roughly 15.0% to 20.0% of dementia cases in North America and Europe with somewhat higher estimates of around 30.0% in Asia and developing countries.^2,28,29,30^ Conversely, developing societies, such as Ghana, where hypertension is the major problem, seem to have a proportionally high prevalence of vascular dementia.^31^ This was evident in this study in which hypertension was the commonest comorbidity among patients with dementia. Hypertension, particularly midlife high blood pressure, has been related to a higher risk of cognitive decline and dementia, including Alzheimer’s disease.^32^

### Descriptive statistics of problem area score, met needs and unmet needs

Among the problem areas, 92.0% of carers assessed ‘legal issues’ as no/mild problem. Participants in a previous study reported difficulty in providing adequate care because of privacy laws inherent in healthcare.^33^ In this study, although 94% of carers have received a formal education, problems that were reported as severe included ‘lack of information about dementia’, ‘problems caused by crises’ and ‘lack of information about treatment’. This supports the reports of inadequate knowledge of dementia in sub-Saharan Africa and Asia compared with European populations.^34,35^ This has important implications; it calls for intensive public awareness creation and provision of information on dementia to caregivers, aimed at bridging the knowledge gap and equipping carers to handle crisis situations. In view of the carers’ lack of information on dementia, the study participants’ commonest unmet needs were ‘printed information material’ and ‘hotline where the carer can get advice in crises’.

The met need with the highest proportion (42%) of carers was ‘diagnosis or treatment of the carer by a general practitioner’. This highlights the important role played by primary care physicians and the fact that carers substantially need everyday help from trained professionals,^36^ especially at the early stage of dementia.^37^

Sociocultural nuances apparently influence carers’ assessment of met and unmet needs. As the African (Ghanaian) population is largely communal and supportive of each other, ‘general assistance for household chores’ was reported as the second highest met need by study participants. This contrasts with the findings in a study in Greece (a European society that is relatively less communal) where receiving help at home was the unmet need among a high percentage of caregivers.^38^ Furthermore, in Italy, ‘counselling and emotional support’ was reported as the intervention frequently needed by carers of PWD, and ‘disappointment caused by the illness, concerns about the patient’s future’ was the commonest problem.^7^

### Correlation of demographic characteristics, problem area score, met needs and unmet needs

The study found that the age of a carer was negatively correlated with the number of unmet needs. This compares with a similar study that found a statistically significant relationship with age and moderate to high unmet needs.^39^ Younger carers recorded higher numbers of unmet needs compared with older carers. It is, therefore, imperative for age to be factored into policies and interventions to reduce the burden of carers of PWD.

The authors also found a statistically positive correlation between the problem area score and the number of unmet needs. This implies that as the assessment of problem severity increases, the total number of unmet needs also increases. Previous studies also reported that higher severity of problems not solved increases the total number of unmet needs with an increasing burden on the carer.^6,7,40^ This may heighten their risk of suffering mental disorders such as depression and anxiety.^7^ However, this was not assessed in the studies participants.

### Strengths and limitations

This multicentre study among a diverse group of carers of PWD in Ghana using the CNA-D tool is a significant contribution to the much-needed literature on challenges associated with dementia, which is a looming epidemic in sub-Saharan Africa. Following up on previous studies in more advanced countries, the authors have highlighted the problems faced by carers of PWD with varying levels of severity and identified their met and unmet needs peculiar to a population living in low- and middle-income countries. The data were assessed on only the carers’ needs. Hence, the authors were unable to draw correlations between those needs and the functional status of the people with disabilities. The authors were limited by its cross-sectional design that did not allow for the provision of intervention to problems identified and unmet needs. The authors recommend that future studies should consider a before-and-after assessment of needs among two different groups of carers exposed to specific interventions.

## Conclusion

Family members constitute a significant proportion of carers of PWD in Ghana. Majority identify a lack of education on dementia and limited access to information on the disease as a severe problem. They, however, consider diagnosis or treatment by a general practitioner as a met need. Younger carers and those who provided higher scores of problem severity identified more unmet needs. The authors call for increased public education and availability of information materials on dementia with a focus on families with PWD.
